# Effect of Endosymbiotic Bacteria on Fungal Resistance Toward Heavy Metals

**DOI:** 10.3389/fmicb.2022.822541

**Published:** 2022-03-15

**Authors:** Simone Lupini, Janire Peña-Bahamonde, Gregory Bonito, Debora F. Rodrigues

**Affiliations:** ^1^Department of Civil and Environmental Engineering, University of Houston, Houston, TX, United States; ^2^Department of Biology and Biochemistry, University of Houston, Houston, TX, United States; ^3^Department of Plant, Soil, and Microbial Sciences, College of Agriculture and Natural Resources, Michigan State University, East Lansing, MI, United States

**Keywords:** endobacteria, heavy metals, fungi, metal removal, adsorption, host resistance

## Abstract

Most studies on metal removal or tolerance by fungi or bacteria focus on single isolates, without taking into consideration that some fungi in nature may be colonized by endobacteria. To address this knowledge gap, we investigated the tolerance and removal of diverse metals with two fungal species: *Linnemannia elongata* containing Burkholderia-related endobacteria and *Benniella erionia* containing Mollicute-related endobacteria. Isogenic lines of both species were generated with antibiotic treatments to remove their respective endobacteria. Experiments involved comparing the isogenic lines and wild type fungi in relation to the minimum inhibitory concentration for the metals, the fungal ability to remove these different metals *via* atomic adsorption spectroscopy, and the interaction of the metals with specific functional groups of the fungi and fungi-bacteria to determine the role of the bacteria *via* attenuated total reflection fourier transformed infrared (ATR-FTIR). Finally, we determined the influence of different metal concentrations, associated with moderate and high fungal growth inhibition, on the presence of the endobacteria inside the fungal mycelium *via* quantitative real-time PCR. Results showed that the presence of the endosymbiont increased *B. erionia* resistance to Mn^2+^ and increased the removal of Fe^2+^ compared to isogenic lines. The absence of the endosymbiont in *L. elongata* increased the fungal resistance toward Fe^2+^ and improved the removal of Fe^2+^. Furthermore, when the bacterial endosymbiont was present in *L. elongata*, a decrease in the fungal resistance to Ca^2+^, Fe^2+^, and Cr^6+^was noticeable. In the ATR-FTIR analysis, we determined that C-H and C = O were the major functional groups affected by the presence of Cu^2+^, Mn^2+^, and Fe^2+^ for *L. elongata* and in the presence of Cu^2+^ and Ca^2+^ for *B. eronia*. It is noteworthy that the highest concentration of Pb^2+^ led to the loss of endobacteria in both *L. elongata* and *B. eronia*, while the other metals generally increased the concentration of endosymbionts inside the fungal mycelium. From these results, we concluded that bacterial endosymbionts of fungi can play a fundamental role in fungal resistance to metals. This study provides the first step toward a greater understanding of symbiotic interactions between bacteria and fungi in relation to metal tolerance and remediation.

## Graphical Abstract

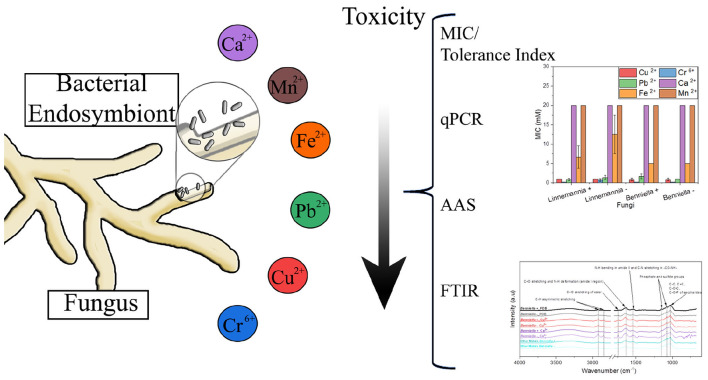

## Introduction

Fungi and bacteria are known for their resistance toward metals (Zafar et al., 2007; Aguirre and Culotta, 2012; Lisher and Giedroc, 2013; Kumar and Dwivedi, 2021). However, most studies regarding the ability of fungi and bacteria to resist and remove metals from the environment are still focused on pure cultures and do not take into consideration the impacts of symbionts. In the environment, fungi and bacteria take part in a wide range of biogeochemical cycles, with consequent formation of intimate relationships. In fact, in recent years fungi have also been characterized for their capacity to harbor bacteria in their microbiome, both inside and outside (Robinson et al., 2021). However, little is known about the functionalities of these relationships, or how the external environment impacts these interactions.

Metals are important elements in the environment that can directly impact the survival of diverse organisms. Metals can be classified as non-essential or essential based on their positive or negative interactions with living organisms (Gadd, 1994) and their long-term effects on biological systems (Rainbow, 1995; Appenroth, 2010). Essential and non-essential metals at different concentrations can be found depending on the location, e.g., proximity to mining (Navarro et al., 2008), agriculture (Vaalgamaa and Conley, 2008), or other industries (Cortes et al., 2003). Furthermore, metals do not biodegrade; they can only be extracted or transformed (ul Hassan et al., 2017).

Non-essential metals, commonly called heavy metals, are among the environmental contaminants most affecting the balance of ecosystems (Smejkalova et al., 2003). Unlike essential metals (including Ca^2+^, Mn^2+^, and Fe ^2+^), which take part in various biological processes as micronutrients and co-factors of enzymes (Tebo et al., 2005), heavy metals (including Cu^2+^, Cr^6+^, and Pb^2+^) are characterized by a broad range of cytotoxicity. In general, all metals, essential or not, can be toxic to microorganisms depending on their concentration. For this reason, different biological systems have evolved different mechanisms to mitigate their toxicity (Temple and Le Roux, 1964; Bitton and Freihofer, 1977; Cervantes and Gutierrez-Corona, 1994). Fungi tend to be the most resistant to metals compared to bacteria and other microorganisms (Mejias Carpio et al., 2018). The innate ability of fungi to resist heavy metals has been studied and is considered a sustainable approach for remediation processes (Johnson and Choudhary, 2016; Cecchi et al., 2019; Qin et al., 2020; Gunjal, 2021; Kumar and Dwivedi, 2021; Neogi et al., 2021; Tomer et al., 2021). However, the possibility that fungal resistance to metals may be influenced by the presence of endobacteria has not been considered previously.

This study aimed to determine whether the presence of intracellular bacterial symbionts of fungi influence the response of their host to different types and concentrations of metals. For this purpose, two fungi, *Linnemannia elongata* (NVP64) and *Benniella erionia* (GBAus27b), previously determined to harbor endobacteria, were selected as candidates in this present study (Uehling et al., 2017; Desirò et al., 2018). Isogenic lines of both species were generated with antibiotic treatments to remove their respective endobacteria and serve as control treatments. We tested the innate metal tolerance and capacity to remove the metals by these two fungal species with and without endosymbionts through Minimum Inhibitory Concentration, Tolerance Index, Atomic Absorption Spectroscopy (AAS), and Fourier Infrared Spectroscopy. This study offers a broader view of the impact of impending metal contamination on the tolerance and survival of fungi, the role of bacterial endosymbionts in fungi on the metal resistance, and the part that diverse types of metals may exert, as environmental stressors, to fungal microbiomes.

## Materials and Methods

### Media and Solution Preparations

Separate metal stock solutions containing 0.1 M of Cu^2+^, Cr^6+^, Ca^2+^, Pb^2+^, Mn^2+^, and Fe^2+^ were prepared by dissolving the following salts in distilled water (DIW) followed by filter sterilization [0.2 μm Polyethersulfone membrane filter (Thermo Fisher Scientific)], e.g., copper sulfate (CuSO_4_), chromium oxide (CrO_3_), lead nitrate [Pb (NO_3_)_2_], calcium chloride (CaCl_2_), manganese sulfate (MnSO_4_), and iron sulfate (FeSO_4_). The media used to grow the fungi were Potato Dextrose Broth (PDB) and Potato Dextrose Agar (PDA); both were purchased from Sigma-Aldrich. The pH of the media was adjusted to pH ≈5.6 with either 1 M NaOH or 1 M HCl (Hitchins et al., 1998), and autoclaved at 121°C for 15 min. The sterilized media was then supplemented with the sterile metal stock solution to obtain the appropriate final concentration of the metal (Zhang et al., 2020). All the reagents were purchased from Sigma Aldrich and were used as received.

### Fungal Isolates and Growing Conditions

The fungal cultures used in this study were *Linnemannia elongata* (NVP64), previously characterized for the presence of *Burkholderia*-related endosymbiont (BRE) (Uehling et al., 2017), and *Benniella erionia* (GBAus27b), characterized for Mollicute-related endosymbiont (MRE) (Desirò et al., 2018). Both species were investigated with cultures containing their respective endobacteria, denoted as wild-type, and isogenic lines that were “cured” from their endobacteria through antibiotic treatments (Uehling et al., 2017).

The successful removal of MRE and BRE with antibiotics to generate endobacterial-free isogenic fungal lines was confirmed by TEM and qPCR in previous publications (Uehling et al., 2017; Desirò et al., 2018). We used these same isogenic lines. All isolates are maintained on antibiotic-free media, and have been for years, and experiments were carried out in antibiotic-free media. Thus, it is unlikely that antibiotic treatments impacted the presented data. For simplification, in the present study, we will refer to *L. elongata* NVP64 as *Linnemannia* and *B. erionia* GBAus27b as *Benniella*. Also, the wild-type (WT) strains will be named as either *Linnemannia*+ or *Benniella* +, and control isogenic fungi lacking endobacteria will be abbreviated as *Linnemannia* – or *Benniella* –. The isogenic lines were also tested for the presence/absence of the bacteria signal prior to the start of the experiments in the present study (data not shown).

### Minimum Inhibitory Concentration and Tolerance Index

The tolerance of chosen fungal isolates toward heavy metals was tested by assessing the minimum inhibitory concentration (MIC) (Zafar et al., 2007). Different amounts of each metal stock (Cu^2+^, Cr^6+^, Ca^2+^, Pb^2+^, Mn^2+^, and Fe^2+^) were added to the PDA culture media, to obtain the desired final concentrations in the range of 0.1–20 mM. Each plate was prepared in triplicate and subsequently divided into four sections of equal size. For each of the four isolates, an 8-mm agar plug with mycelium was taken from a 7-day-old pre-grown PDA plate and placed in the center of test plates under sterile conditions. The plates were incubated at 25°C between 2 and 5 days, based on the fungal growth. Three different plates were used for each concentration of the different metals. The diameter of each fungus was monitored for 5 days. The MIC value for each metal was defined as the minimum metal concentration at which no fungal growth was observed on all the replicates (Zafar et al., 2007). As a control, the growth of the fungi was also monitored in the presence of PDA media, not supplemented with metals.

Once determined the MIC value for each metal, the fungi were grown in PDA plates amended with the metal at a final concentration corresponding to a visible inhibition (slightly lower than the MIC value) (0.5 mM Cu^2+^, 0.1 mM Cr^6+^, 20 mM Ca^2+^, 0.5 mM Pb^2+^, 20 mM Mn^2+^, and 2 mM Fe^2+^) and incubated at 25°C. After 3 days of growth, the diameter of the fungal mycelium was measured to determine the tolerance index (TI). The TI can be defined as the ratio between the diameter of the fungus in the presence of metals and its control without any metals (Joo and Hussein, 2012).

### Endobacteria Quantification: DNA Extraction and Quantitative Polymerase Chain Reaction

The quantification of endobacteria was determined *via* quantitative polymerase chain reaction (qPCR) after the exposure to two different metal concentrations, e.g., visible inhibition and non-visible inhibition (Uehling et al., 2017; Desirò et al., 2018). In the present study, visible inhibition was defined as the concentration, below the MIC value, at which the growth of the fungus was still possible. Non-visible inhibition was defined as an intermediate concentration of metals between MIC and the absence of metal, characterized by a negligible inhibition of the fungal growth compared to the control. These conditions have been chosen to determine if, at different degrees of fungal growth inhibition by the metals, there were changes in the endobacteria concentration. To investigate that, 8-mm agar plugs containing 7-day-old fungal mycelium were added to flasks containing 100 ml of PDB media supplemented or not with metals (Cu^2+^: 0.5 and 0.01 mM; Cr^6+^:0.1 and 0.05 mM; Ca^2+^: 20 and 10 mM; Pb^2+^:0.5 and 0.01 mM; Fe^2+^: 2 and 1 mM; and Mn^2+^: 20 and 10 mM). The flasks were kept for 5 days at 25°C at constant shaking. Then, the biomass was separated from the supernatant and weighted, and 100 mg of grown biomass was added in a tube. The extraction was carried using the Zymo extraction kit (Zymo Quick-DNA Fungal/Bacterial Kit, D6005). The extracted DNA was checked for quality control with a microplate reader (Take3, BioTek Instruments, Winooski, VT, United States) to evaluate the DNA concentration and degree of purity (260/280 nm ratio).

The PCR mix was prepared following the protocol for the PowerUp SYBR Green Master Mix (Applied Biosystems) (Mix, 2011). The primers used for this study were E8-F and E533-R (Nguyen et al., 2017). The quantification of the bacteria in the fungal isolates was performed on a StepOnePlus (Applied Biosystems) qPCR machine using the following protocol: enzyme activation at 95°C for 10 min, followed by 40 cycles of denaturing at 95°C for 15 s, and annealing at 60°C for 1 min. The melting curve was also monitored to determine non-specific amplification. The endobacterial quantification was estimated by comparing the *C*_t_ value with the standard curve obtained from serial dilutions of *E. coli* K12 genomic DNA (*R*^2^ = 0.9948, Supporting information Supplementary Figures 1, 2) as previously described (Lee et al., 2008). The gene copy was normalized by nanograms of DNA and grams of biomass.

### Quantification of Metal Removal *via* Flame Atomic Adsorption Spectroscopy

To investigate the metal removal, 8-mm agar plugs containing the fungal mycelium were transferred to flasks containing 100 ml of PDB media supplemented with the metals, e.g., 0.5 mM Cu^2+^, 0.1 mM Cr^6+^, 20 mM Ca^2+^, 0.5 mM Pb^2+^, 20 mM Mn^2+^, and 2 mM Fe^2+^. Positive and negative controls for this experiment were also evaluated and included sterile metal-free medium, sterile metal-added medium, and fungus grown in absence of metal. The flasks were kept for 5 days at 25°C at constant shaking at 125 rpm. The supernatant and biomass were separated by filtration using a 0.45 μm PES (Polyethersulfone) membrane filter (Thermo Scientific), and then the supernatant was transferred to a clean sterile tube for further analysis. The quantification of metal biosorption by the different fungi was evaluated using flame atomic absorption spectroscopy (AAS) (AAnalyst 200, Perkin Elmer) with Cu^2+^, Cr^6+^, Ca^2+^, Pb^2+^, Mn^2+^, and Fe^2+^ lamps from Perkin Elmer. To determine if the removal was due to metabolic processes, the adsorption of the metals to the mycelium was also performed using dead fungal biomass. For the dead fungal biomass assay, the fungi were grown for 5 days in liquid culture and subsequently autoclaved for 30 min at 121°C and 103 kPa. The same weight of mycelium obtained in the previous experiment was introduced as dead biomass to reduce the variability between the two experiments. Then, the culture obtained was incubated for 1 day at 25°C in the presence and absence of metals (0.5 mM Cu^2+^, 0.1 mM Cr^6+^, 20 mM Ca^2+^, 0.5 mM Pb^2+^, 20 mM Mn^2+^, and 2 mM Fe^2+^). After that, the supernatant obtained from the liquid culture was filtered using 0.2 μm PES (Polyethersulfone) syringe filters (Thermo Scientific), diluted based on the range of optimal concentrations for the lamps (0.03–2 ppm for Cu^2+^, 0.1–5 ppm for Cr^6+^, 0.1–5 ppm for Ca^2+^, 0.2–10 ppm for Pb^2+^, 0.2–7 ppm for Mn^2+^, and 0.01–3 ppm for Fe^2+^). The solutions relative to each experiment were amended with HNO_3_ to obtain a 2% final concentration of the acid before being analyzed. A seven-point standard curve was prepared for each of the elements analyzed. For Cu^2+^, we used seven different concentrations in the range 0.5–15 ppm, for Cr^6+^ from 0.5 to 10 ppm, for Pb^2+^ from 0.02 to 30 ppm, for Ca^2+^ from 0.5 to 10 ppm, for Fe^2+^ from 0.5 to 20 ppm, and for Mn ^2+^ from 0.5 to 20 ppm. Then, the absorbance of each of the different metals was interpolated in the calibration curve to determine the residual metal concentrations in the solution.

Each experiment was conducted in triplicates, and the obtained mean for each condition was compared to the respective control metal-containing media to determine the percentage of removal. A Student’s *t*-test was also performed to determine if the means of the values were statistically significant.

### Morphological Analysis of the Functional Groups With Fourier Transformed Infrared Spectroscopy

The effect of the biosorption of the different metals toward the physiochemical properties of the fungi was evaluated *via* Fourier Infrared Spectroscopy (FTIR) Digilab FTS 7000 equipped with an HgCdTe detector analysis and combined with Attenuated Total Reflection (ATR). For the analysis, the biomass obtained after the incubation (5 days at 25°C at constant shaking) of flasks containing 100 ml of PDB media supplemented with metals (0.5 mM Cu^2+^, 0.1 mM Cr^6+^, 20 mM Ca^2+^, 0.5 mM Pb^2+^, 20 mM Mn^2+^, and 2 mM Fe^2+^) with 8-mm agar plugs of the fungal mycelium grown for 7 days on a plate was separated by filtration using a 0.45-μm PES (Polyethersulfone) membrane filter (Thermo Fisher Scientific) from the supernatant. A control was also prepared by inoculating the media without any metals. Approximately, 0.5 g of biomass obtained from the liquid culture was collected, transferred to a petri dish, and dried at room temperature under the biohood until completely dry. The dry mycelium was transferred using tweezers with the mycelium facing down and scanned in the medium range (4,000–670 cm^–1^) with a 4 cm^–1^ resolution. The data from the ATR-FTIR was processed using the R package Chemospec (Lucas, 2006; Hanson, 2014).

### Statistical and Data Analysis

All the experiments reported were carried out in triplicate. The averages and standard deviations of triplicate measurements were reported for all the experiments. Statistical analysis was carried out using Excel (Microsoft Corporation, Redmond, WA, United States), R studio, and Origin (OriginLab Corporation, Northampton, MA, United States).

The ATR-FTIR spectra were normalized based on the most intense peak (1,030 cm^–1^) and loaded in R-Studio. Using the R-package ChemoSpec (Hanson, 2014), the region with no peaks was removed (1,900–2,600 cm^–1^) using the command “removeFreq” to reduce the noise. After the removal of the regions with no interest, the hcaSpectra command was used to obtain the Euclidean distance between the samples and plot the Principal Component Analysis (PCA) and Hierarchical Cluster Analysis (HCA) results (Varmuza and Filzmoser, 2016).

## Results

### Determination of Minimum Inhibitory Concentration

**FIGURE 1:**
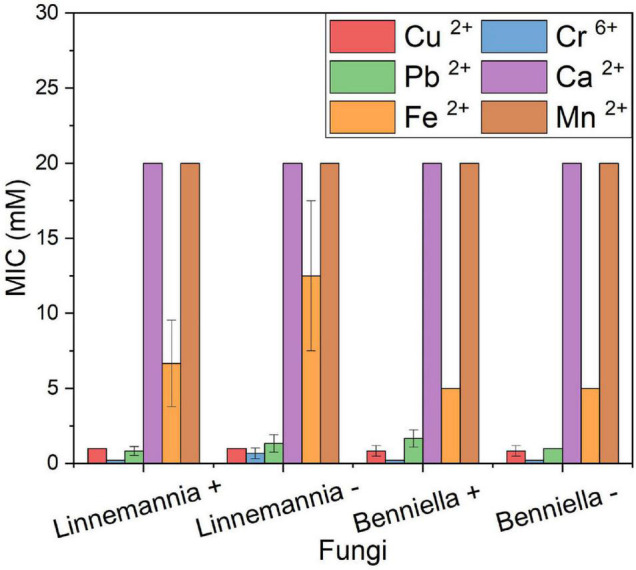
MIC of heavy metals of the WT strains (+) and cured (–) fungi *Benniella* - and *Linnemannia* -. The fungi were grown in PDA plates supplemented with different metal concentrations (range, 0.1–20 mM). The growth at 25°C was monitored for up to 5 days to determine the minimum concentration of the metal that completely inhibited the fungal growth. Statistically significant differences between WT strains (+) and cured (–) were evaluated using the Student’s *t*-test. No statistically significant difference was found between the different isogenic fungi.

The resistance against six different metal ions of the two fungi, *L. elongata* (*Linnemannia*) and *B. erionia* (*Benniella*) is reported as MICs in Figure 1. In the presence of concentrations as high as 5 mM for Cu^2+^, Cr^6+^, and Pb^2+^, both fungi displayed a complete inhibition, while for Fe^2+^, the maximum MIC concentration was 10 mM. The presence of Ca^2+^ and Mn^2+^ in the media did not inhibit the fungal growth, even at concentrations as high as 20 mM. From the comparison of the MIC between cured and the wild type, the absence of the endobacteria seemed to have promoted the resistance of *L. elongata* toward Pb^2+^, Cr^6+^, and Fe^2+^ while an inverted trend was observed for the fungus *B. erionia*. Clearly, the presence of endobacteria had different effects on different fungi.

### Tolerance Index

**FIGURE 2:**
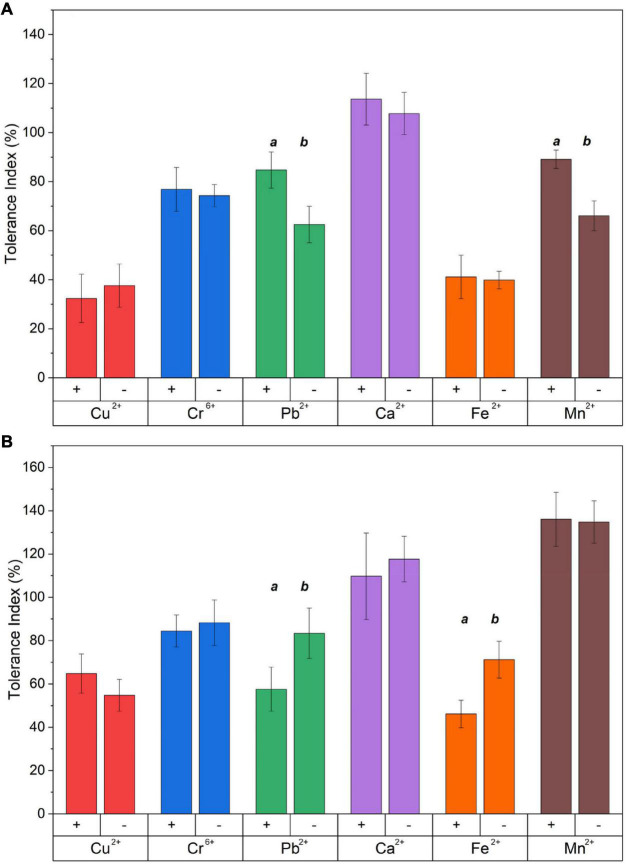
Tolerance index of **(A)** *Benniella* and **(B)** *Linnemannia* in the presence of different metals. The fungi were grown in PDA plates supplemented with different metals (0.5 mM Cu^2+^, 1 mM Cr^6+^, 20 mM Ca^2+^, 0.5 mM Pb^2+^, 20 mM Mn^2+^, and 2 mM Fe^2+^). The diameter of the fungal mycelium after 3 days was compared to the control (PDA) without metal. Statistically significant differences between WT strains (+) and cured (–) fungi were evaluated using the Student’s *t*-test. The significance among the samples was assessed using the Student’s *t*-test and reported as alphabet letters. Same letters were attributed for *p*-values > 0.05, while different letters were attributed to *p*-values < 0.05. The lack of letters means that the results were not statistically significant.

The tolerance index, calculated as the ratio between the radial growth of the treated fungus to their respective control, was determined based on the concentrations obtained through the MIC assays, as shown in Figure 2. The percentage reduction of the tolerance index was calculated based on the control without metal. In the presence of Cu^2+^, the fungus *Benniella* exhibited a decrease in tolerance of approximately 40% with no statistically significant difference between *Benniella*+ and *Benniella* –. The occurrence of the endobacteria for the fungus *L. elongata* was beneficial regarding the presence of Cu^2+^ in the media. Compared to the control, *L. elongata* – had a reduction in the tolerance index of 45%, while *Linnemannia*+ had 35%. When exposed to Cr^6+^ the fungus *Benniella* showed a reduction of the tolerance index of approximately 25%, for both *Benniella* – and *Benniella*+, with no statistical significance between the fungi with or without endobacteria. For the *Linnemannia* fungus, in the presence of Cr^6+^, a reduction of approximately 15% with no statistically significant difference was observed for both *Linnemannia* + and *Linnemannia* -. The presence of Pb^2+^ displayed a greater inhibitory effect in the *Benniella* fungi with a reduction of the tolerance index of 15–37% for *Benniella* + and *Benniella* -, respectively. Opposite results were noticed for the fungus *Linnemannia* in the presence of lead, where the tolerance index of *Linnemannia*+ decreased by 42% while *Linnemannia* – 17%. In the presence of Ca^2+^, both *Benniella* and *Linnemannia* performed better than their control media without this metal supplement, with an average increase tolerance index of 12%. Fe^2+^ led to a tolerance index reduction of about 60% for both *Benniella* – and *Benniella*+, with no statistical significance between the two. For *Linnemannia*, the presence of Fe^2+^ in the media reduced the tolerance index by 54% for *Linnemannia*+ and by 29% for *Linnemannia* –. Furthermore, both *Benniella* - and *Benniella*+ were affected by the presence of Mn^2+^, with a respective tolerance reduction of 11 and 34%, while the growth of *Linnemannia* was improved by about 35%. The comparison between *Benniella*+ and *Benniella* – showed that the presence of the endobacteria improved the tolerance of the fungus toward the metals and, particularly, toward Pb^2+^ and Mn^2+^. For *Linnemannia*+ and *Linnemannia* –, the presence of the endobacteria enhanced the fungal resistance only in the presence of Cu^2+^, but not for the other metals.

### Metal Removal Quantification by Flame Atomic Absorption Spectroscopy

**FIGURE 3:**
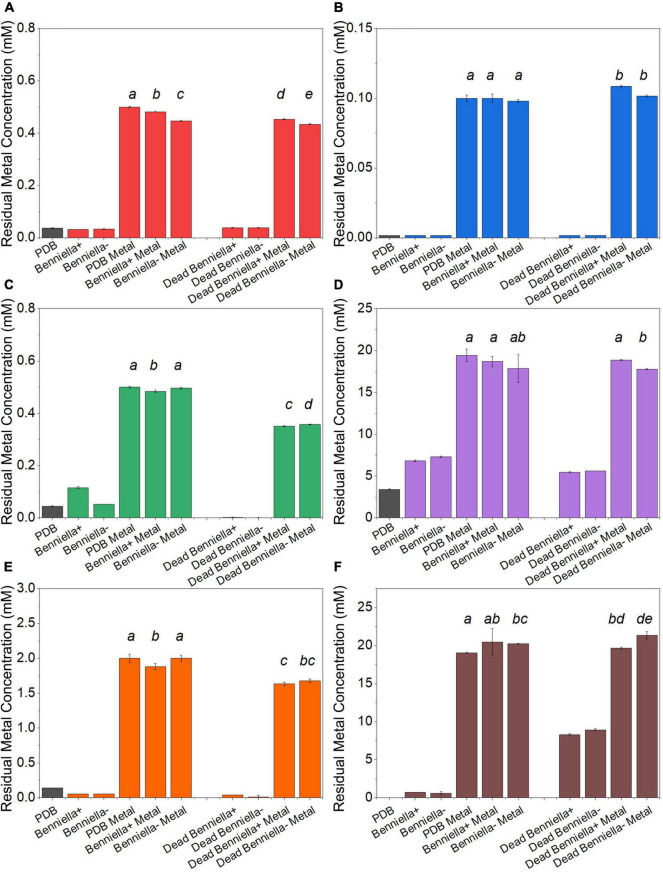
Residual metal concentration by dead and live *Benniella* + and *Benniella* - in the presence of different metals: **(A)** Cu^2+^, **(B)** Cr^6+^, **(C)** Pb^2+^, **(D)** Ca^2+^, **(E)** Fe^2+^, and **(F)** Mn^2+^. The living fungi were grown on PDB supplemented with different metal concentrations (0.5 mM Cu^2+^, 1 mM Cr^6+^, 20 mM Ca^2+^, 0.5 mM Pb^2+^, 20 mM Mn^2+^, and 2 mM Fe^2+^) and incubated for 5 days at 25°C under constant shaking. The dead fungus was added after sterilization and incubated for 1 day at 25°C under constant shaking. Controls for this experiment included metal-free medium, metal-added medium, and fungus grown in the absence of metal. The significance among the samples was assessed using the Student’s *t*-test and reported as alphabet letters. Same letters were attributed for *p*-values > 0.05, while different letters were attributed to *p*-values < 0.05. No letters mean that they were not statistically significant at all. Following the qPCR analysis, it emerged that in presence of Pb^2+^ at 0.5 mM, the presence of the endobacterium was no longer detectable inside the host at the end of the experiment.

**FIGURE 4:**
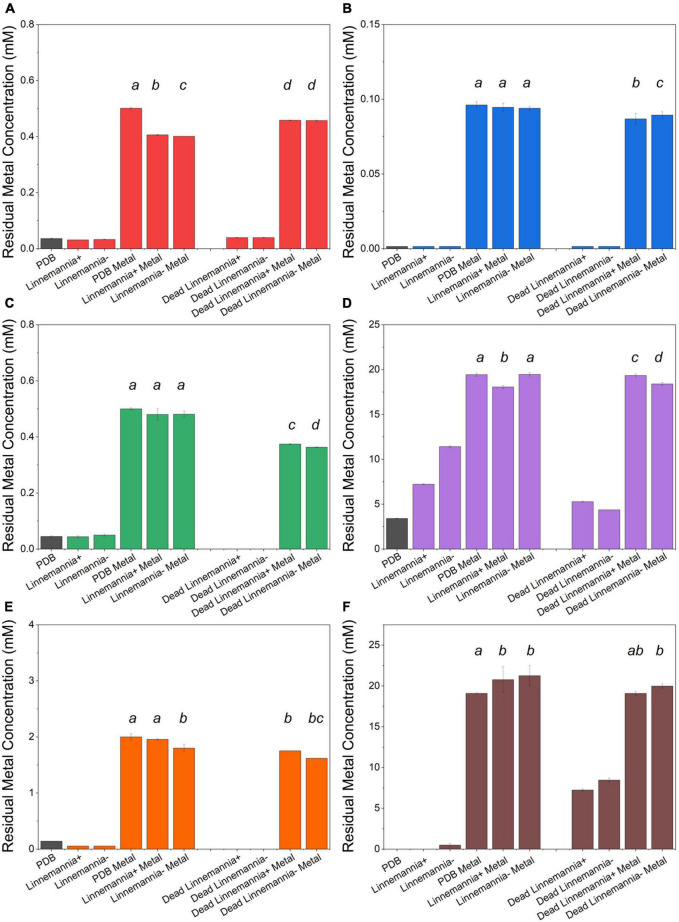
Residual metal concentration by dead and live *Linnemannia* + and *Linnemannia* - in the presence of different metals: **(A)** Cu^2+^, **(B)** Cr^6+^, **(C)** Pb^2+^, **(D)** Ca^2+^, **(E)** Fe^2+^, and **(F)** Mn^2+^. The living fungi were grown on PDB supplemented with different metal concentrations (0.5 mM Cu^2+^, 1 mM Cr^6+^, 20 mM Ca^2+^, 0.5 mM Pb^2+^, 20 mM Mn^2+^, and 2 mM Fe^2+^) and incubated for 5 days at 25°C under constant shaking. The dead fungus was added after sterilization and incubated in the same conditions as the samples. Controls for this experiment included metal-free medium, metal-added medium, and fungus grown in the absence of metal. The significance among the samples was assessed using the Student’s *t*-test and reported as alphabet letters. Same letters were attributed for *p*-values > 0.05, while different letters were attributed to *p*-values < 0.05. No letters mean that they were not statistically significant at all. Following the qPCR analysis, it emerged that in presence of Pb^2+^ at 0.5 mM, the presence of the endobacterium was no longer detectable inside the host.

The metal removal by the fungal biomass was investigated as shown in Figures 3, 4. The figures represent the residual metal concentrations in the solution for each metal evaluated. In relation to the toxic metals, we observed different removal patterns for the different fungi. In the presence of Cu^2+^, the *Benniella* – exhibited almost three times more metal removal than *Benniella*+ (3.6% for *Benniella* – and 10% for *Benniella*+), while the dead fungi were able to adsorb 9 and 13% of Cu^2+^, respectively. *Linnemannia* also exhibited the ability to remove Cu^2+^, approximately 20% for both *Linnemannia*+ and *Linnemannia* –, while the dead mycelium removed roughly 9% with no statistical significance between cured and non-cured strains. The fungus *Benniella* displayed no removal for Cr^6+^, while the dead mycelium, for both *Benniella*+ and *Benniella* –, removed less than 10%. A similar trend was also observed for the fungus *Linnemannia* in the presence of Cr^6+^.

Regarding essential metals, we also observed different behaviors in relation to metal removal for the different fungi investigated. When the fungi were inoculated with Ca^2+^, the results showed that *Benniella* - removed approximately two times more compared to *Benniella*+ (4% for *Benniella*+, 8% for *Benniella* –, 3% for dead *Benniella*+, 9% dead *Benniella* –). The highest removal for the fungus *Linnemannia* coincided with the dead mycelium, with a percentage removal of over 11%. Furthermore, the living *Linnemannia* + removed only 7% of the Ca^2+^, while *Linnemannia* - had no difference compared to the control. In the presence of Fe^2+^, no removal was observed for *Benniella* –. On the other hand, for *Benniella* +, this fungus adsorbed 6% when dead, while the removal for both *Benniella* – and *Benniella* + was over 15%. *Linnemannia in* the presence of Fe^2+^, showed an inverted trend, compared to *Benniella*. Around 5% more removal was observed when *Linnemannia -* was used, compared to *Linnemannia*+. In the presence of Mn^2+^, the supernatant of both *Linnemannia* and *Benniella*, dead and alive, was characterized by having a higher concentration compared to the control, probably due to the release of the manganese from the biomass to the supernatant.

From the comparison between *Benniella*+ and *Benniella* –, the absence of the endobacteria did not appear to affect the overall capacity of the fungus to remove the metals, apart from Fe^2+^, where the presence of the endobacteria enhanced the removal of the metals. A different trend was seen from the comparison of *Linnemannia*+ and *Linnemannia* –, where the presence of the endobacteria did not improve the removal of the metals, except for Ca^2+^. The effect of the inactivation of the fungi was associated with an overall improved metal removal, especially evident in the presence of Pb^2+^ for both *Benniella* and *Linnemannia*.

### Effect on the Abundance of Bacterial Endosymbionts Presence on the Fungal Host Exposure to Metals

**FIGURE 5:**
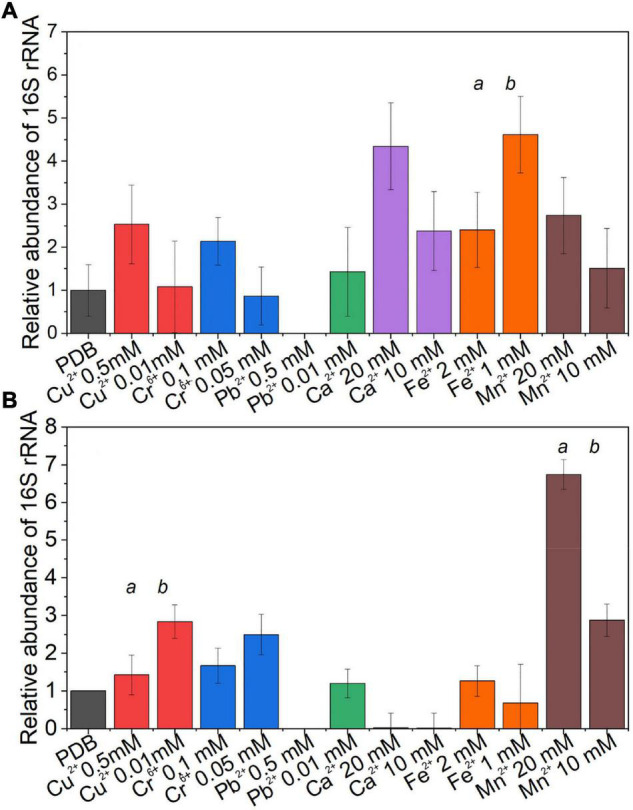
Relative abundance of the bacterial 16S rRNA gene in the fungus **(A)** *Benniella* and **(B)** *Linnemannia* in response to metal presence. The fungi WT were grown in PDB supplemented with two metal concentrations, where visible and non-visible inhibition growth was observed. The fungi were incubated for 5 days at 25°C under constant shaking. An equal amount of mycelium was processed for DNA extraction and qPCR amplification. The significance among the samples was assessed using the Student’s *t*-test and reported as alphabet letters. Same letters were attributed for *p*-values > 0.05, while different letters were attributed to *p*-values < 0.05. No letters mean that they were not statistically significant at all.

The effect of different concentrations of metals on the endobacteria presence in the mycelium was evaluated by comparing the relative quantity of the bacterial 16S rRNA gene at the visible inhibition in the MIC, non-visible inhibition, and the media without the metal, as control (Figure 5). For *Benniella*, the presence of essential metals, *i.e*., Ca^2+^, Fe^2+^, and Mn^2+^, led to a higher relative abundance of the 16S rRNA gene compared to the control at both visible and non-visible inhibitions. The only exception was for Fe^2+^, in which the highest relative abundance of the 16S rRNA gene coincided (Figure 5A) with the highest metal concentration. Different endobacterial abundance was observed for *Linnemannia* (Figure 5B), where only in the presence of Cu^2+^, Cr^6+^, and Mn^2+^, the bacterial load was higher compared to the control at both visible and non-visible inhibitions. We also noticed that the presence of Cu^2+^, Cr^6+^, and Pb^2+^ at a lower metal concentration in the media coincided with a higher relative abundance of endobacteria. Interestingly, for both *Benniella* and *Linnemannia*, we did not detect any bacterial amplification at the highest concentration of Pb^2+^(0.5 mM).

### Interactions of the Metals With the Surface Functional Groups (Attenuated Total Reflection-Fourier Transformed Infrared) of the Fungi

**FIGURE 6:**
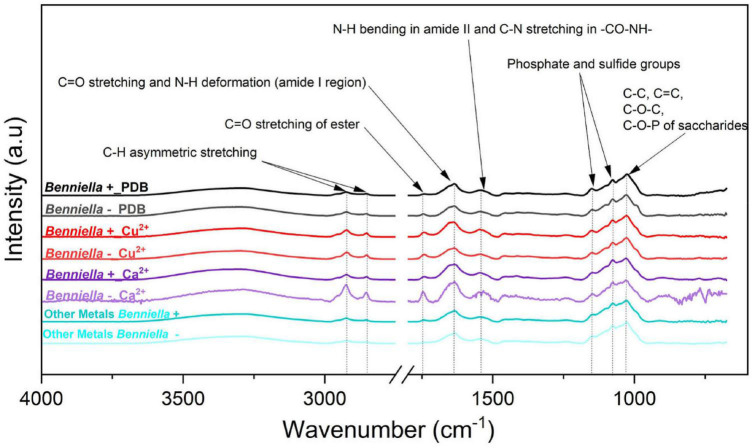
ATR-FTIR spectra of *Benniella* + and *Benniella* - for Ca^2+^, Cu^2+^, and PDB media (control). The spectra of metals Cr^6+^, Pb^2+^, Fe^2+^, and Mn^2+^ had identical peaks; hence, they were merged into “Other metals.” The fungi were grown in PDB added with different metals (0.5 mM Cu^2+^, 1 mM Cr^6+^, 20 mM Ca^2+^, 0.5 mM Pb^2+^, 20 mM Mn^2+^, and 2 mM Fe^2+^) for 5 days at 25°C under constant shaking.

**FIGURE 7:**
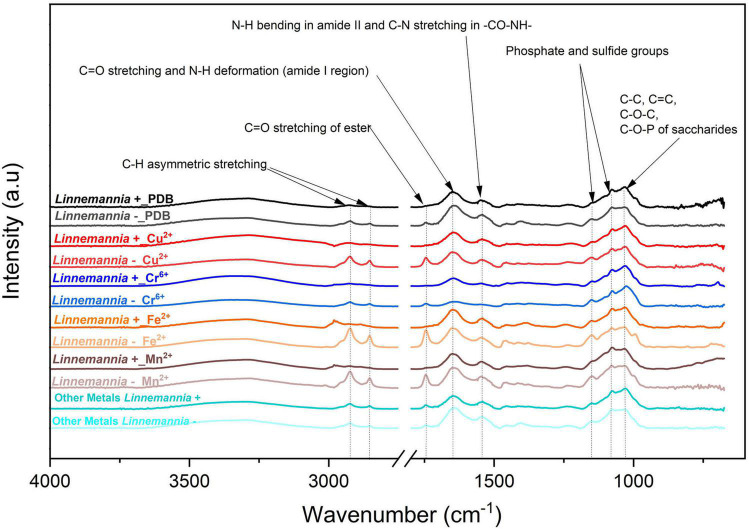
ATR-FTIR spectra of *Linnemannia* + and *Linnemannia* - with PDB, Cu^2+^, Cr^6+^, Fe^2+^, and Mn^2+^. The spectra of metals Pb^2+^and Ca^2+^were identical; hence, they were merged into “Other Metals.” The fungi were grown in PDB added with different metals (0.5 mM Cu^2+^, 1 mM Cr^6+^, 20 mM Ca^2+^, 0.5 mM Pb^2+^, 20 mM Mn^2+^, and 2 mM Fe^2+^) for 5 days at 25°C under constant shaking.

The characterization of the interactions of different metals with the functional groups present on the fungal mycelium was conducted *via* ATR-FTIR analysis. The comparisons of the spectra for *Benniella* and *Linnemannia* are presented in Figures 6, 7, respectively (complete ATR-FTIR spectra of *Benniella* and *Linnemannia*, Supplementary Figures 3, 4, Relative peak intensities, Supplementary Table 1). Both fungi, *Benniella* and *Linnemannia*, showed spectra containing the main functional groups related to proteins and lipids. The spectra showed a broad band in the range 3,000–3,550 cm^–1^, which correspond to the hydroxyl and amino groups (Rao, 1963) present in proteins (Park et al., 2005; Bombalska et al., 2011), followed by two distinct peaks at 2,924 and 2,853 cm^–1^ relative to the asymmetric and symmetric stretching vibration of the -CH present in the lipids (Solomons, 2016). Furthermore, the spectra showed the presence of peaks related to the protein at 1,743, 1,643.6, and 1,546 cm^–1^, associated with the *C* = O stretching, protein amide I, and protein amide II (Kaushik et al., 2010).

The changes observed in the ATR-FTIR for the different fungi were attributed depending on whether they were cured from the bacteria or were exposed to different metals. The ATR-FTIR spectra of the fungus *Benniella* + in the presence of Cu^2+^, Cr^6+^, and Ca^2+^ showed an increased intensity in the C-H stretching from the lipids (2,924 and 2,853 cm^–1^), and the amide from the protein (1,742 cm^–1^). For *Benniella* + in the presence of Pb^2+^ and Fe^2+^, we observed a noticeably decrease in intensity of the peaks associated with the amide group of the protein (1,742, 1,637, and 1,544 cm^–1^) and the C-H of the lipid (2,924 and 2,853 cm^–1^). When the same fungus was grown in the presence of Mn^2+^, a slight decrease in the symmetrical and asymmetrical stretching of PO^2–^ and P (OH)_2_ at 1,150, 1,077, and 1,026 cm^–1^ was observed (Amann et al., 1990; Bombalska et al., 2011) compared to the control without the metal.

In the case of *Benniella* –, when it was in contact with Cu^2+^, Cr^6+^, and Ca^2+^, an increase in the intensity of the C-H stretching from the lipids (2,924 and 2,853 cm^–1^) and the amide from proteins (1,742 cm^–1^) were observed. Especially, the increase in intensity was more notable in the presence of Ca^2+^. However, from Supplementary Table 1, we can see that in the presence of Pb^2+^, Mn^2+^, and Fe^2+^, there was a decrease in intensity of the C-H stretching from the lipids (2,924 and 2,853 cm^–1^), and the amide from the protein (1,742 cm^–1^). The comparison between *Benniella*+ and *Benniella* – showed that the previously discussed peaks, *i*.*e*., lipidic, protein, and phosphate functional groups are enhanced in the presence of endobacteria, indicating that the presence of endobacteria is playing an important role in the presence of those functional groups when the metals are present.

In the case of the fungus *Linnemannia*, the spectra of *Linnemannia*+ in the presence of Cr^6+^ showed a decreased intensity of the amide functional group of the protein (1,742 and 1,544 cm^–1^) compared to the *Linnemannia* without the metal. In the presence of Ca^2+^, an increase of the C-H stretching from the lipids was noticeable when compared to the control with no metal (2,924 and 2,853 cm^–1^) followed by the amide peak of the protein (1,742 cm^–1^). Furthermore, the amide group of the protein was slightly shifted from 1,747 to 1,743 cm^–1^ when *Linnemannia* + was incubated with Ca^2+^. Moreover, in the presence of Fe^2+^ and Mn^2+^, the lipidic peak also shifted to higher wavenumbers, from 2,924 to 2,980 cm^–1^.

For *Linnemannia* –, a sharp increase in peaks associated with the C-H stretching from the lipids (2,924 and 2,853 cm^–1^) and amide functional group from the proteins (1,742 cm^–1^) was noticeable when the fungus was in the presence of Cu^2+^, Fe^2+^, and Mn^2+^. However, in the presence of Cr^6+^, a significant reduction in the functional groups was observed. Furthermore, in the presence of Ca^2+^, the intensity of the peaks associated with the amide functional group of the protein (1,637 and 1,544 cm^–1^) was reduced. Overall, the comparison between *Linnemannia* + and *Linnemannia* - showed that the presence of endobacteria led to stronger intensity and interactions of the functional groups associated with the lipids and proteins with the metals, indicating again that the presence of endobacteria is playing an important role in the presence of lipids and proteins when the metals were present.

**FIGURE 8:**
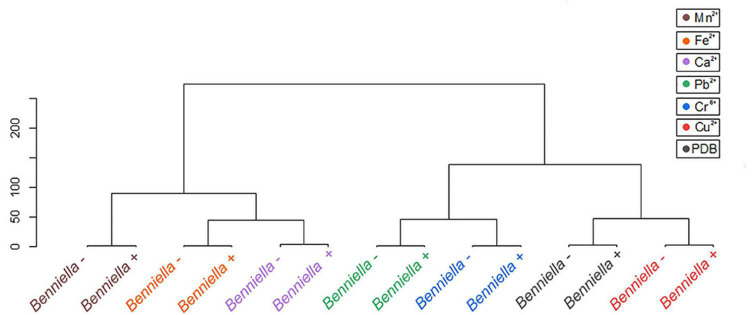
Hierarchical cluster analysis of the ATR-FTIR spectra of the *Benniella* + and *Benniella* –. The Euclidean method was used to determine the distance between the different samples.

**FIGURE 9:**
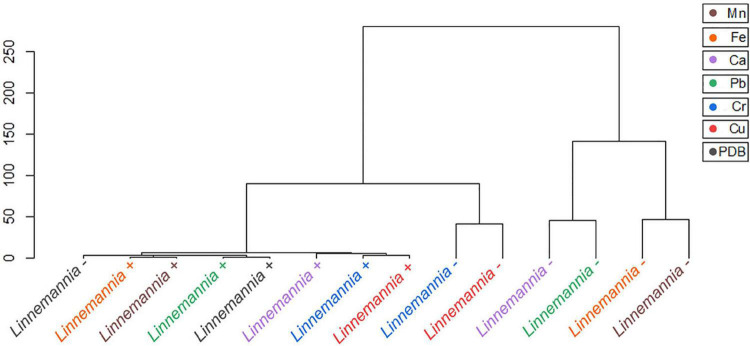
Hierarchical cluster analysis of the ATR-FTIR spectra of the *Linnemannia* + and *Linnemannia* –. The Euclidean method was used to determine the distance between the different samples.

Statistical tools, such as HCA and PCA as shown in Figures 8, 9 and Supplementary Figures 5, 6, were used to assess the interaction between the different functional groups of the fungi and the metals evaluated. The analysis of the *Benniella*+ and *Benniella* – fungi in combination with all the metals evaluated i.e., Mn^2+^, Fe^2+^, Ca^2+^, Cu^2+^, Pb^2+^, and Cr^6+^, showed two different clusters (Figure 8). Those clusters, i.e., Mn^2+^, Fe^2+^, and Ca^2+^ (essential metals), and Cu^2+^, Pb^2+^, and Cr^6+^ (non-essential metals) indicated that the differences observed in the spectra are not related to the presence or absence of endobacteria, but to the type of metal. The type of metals seems to play an important role in the interaction strength between the functional groups and the metals. However, the HCA analysis for *Linnemannia* – and *Linnemannia*+ (shown in Figure 9) indicated that the presence or absence of endobacteria plays a major role in the interaction between the microorganism and the metals. The clusters are clearly based on the presence of endobacteria rather than on the type of metal.

## Discussion

The metals in diverse environments can be classified as essential (e.g., Ca^2+^, Fe^2+^, and Mn^2+^) or non-essential (e.g., Cr^6+^, Cu^2+^, and Pb^2+^) based on their interaction with living organisms (Gadd, 1994). Fungi and bacteria are both important in the biological cycles of metals; however, the effect that the fungal endobacteria can have on resistance and uptake of essential and non-essential metals by the fungal host has not yet been demonstrated.

This study was focused on determining the effects of endohyphal bacteria on the response of fungal hosts to essential and non-essential metals. The fungi *L. elongata* (NVP64) and *B. erionia* (GBAus27b) were selected as candidates for this study for their capacity to harbor BRE and MRE endobacteria, respectively, and for their ability to be cured of their endosymbionts.

The results showed that the two fungal species have different susceptibilities toward certain metals, with Cr^6+^ presenting the highest toxicity toward both fungi, with MIC 0.2 mM for *Benniella* and 0.5 mM for *Linnemannia* – and 0.2 mM *Linnemannia*+, followed by Cu^2+^, 1 mM for both *Benniella* and *Linnemannia*, and Pb^2+^, 2 mM for *Benniella*+, 1 mM for *Benniella* –, 0.5 mM for *Linnemannia*+, and 1 mM for *Linnemannia* –. Regarding the susceptibility of both fungi, independently from the endobacterial presence, non-essential metals such as Cu^2+^, Cr^6+^, and Pb^2+^ had a greater inhibitory effect compared to the other three metals, Ca^2+^, Fe^2+^, and Mn^2+^. For these non-essential metals, the level of inhibition followed this order: Cr^6+^ > Cu^2+^ > Pb^2+^. This result validates the non-essentiality characteristics of Cr^6+^ and Pb^2+^ since these metals are known for their antimicrobial activities, and Cu^2+^, which, although essential, is toxic at high concentrations (Lemire et al., 2013; Mejias Carpio et al., 2018).

**FIGURE 10:**
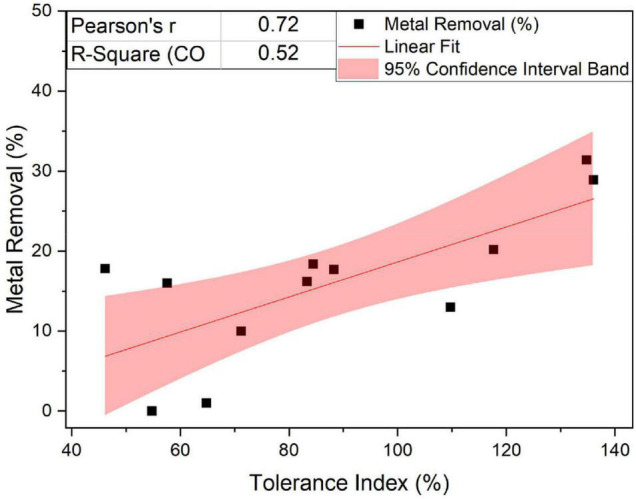
Linear fit of the percentage of removal and metal tolerance of the fungus *Linnemannia*.

Regarding the general performance toward different metals, we observed that the fungus *Linnemannia*, both cured and WT, had an overall higher metal removal capacity than *Benniella*. In fact, although the two fungi are closely related, the associated endosymbionts are phylogenetically distant. Both endosymbionts have a major effect on the metabolism and growth of the fungal host (Li et al., 2017; Uehling et al., 2017), especially in the presence of metals. This was particularly evident for Cu^2+^, Fe^2+^, and Mn^2+^. This result is aligned with the results of the tolerance test, where the *Linnemannia* had a higher tolerance to these metals than *Benniella* (Figure 2). The relationship between the tolerance and the metal removal was further confirmed by linear correlation analysis (Pearson’s R = 0.72) for the fungus *Linnemannia* (Figure 10).

### Endosymbionts Can Affect Differently Fungal Metal Resistance

In the present study, the fungal candidates have been chosen to gain a better understanding of the functionality of the fungal microbiomes in relation to metal tolerance and removal and also to determine the importance of inter-Kingdom cooperation in metal-stressed environments. This work also evaluates the role that endobacteria have on the innate resistance of the fungus for metals known for their toxicity (Cr^6+^, Cu^2+^, and Pb^2+^) and metals, such as Ca^2+^, Fe^2+^, and Mn^2+^ which, although considered essential, can be potentially toxic at high concentrations.

To further gain an understanding of the increase in metal resistance due to the presence of endobacteria, we analyzed the data obtained from the MIC and tolerance index of the two fungi and compared it to the performance of the WT and the fungi cured from the bacteria. Our results show that the metal resistance is influenced by the type of endosymbiont, BRE *vs.* MRE, as well as the type of metals. In the case of the type of endosymbiont, for instance, the presence of the endobacteria in the fungus *Benniella* was clearly beneficial for the fungus with respect to metal tolerance, whether the metal was essential or not. The fungus *Linnemannia*, when cured from its endosymbiont, was more resistant to Fe^2+^, while for the other metals no statistically significant difference was found. This result is surprising since *Burkholderia* species have been described to be resistant to different metals (Caballero-Mellado et al., 2007; Jiang et al., 2008; Schwager et al., 2012; Mullins et al., 2019). In fact, for *Linnemannia*, the presence of this group of bacteria does not appear to be beneficial to the fungi to improve the MIC or the tolerance index toward the presence of metals. This result could be linked to the fact that the maintenance of the endosymbiont leads to a metabolic cost to the host, previously reported to be around 30% (Uehling et al., 2017). Therefore, in the case of *Linnemannia*, the presence of the endobacteria and toxic concentrations of metals might have exacerbated the metabolic cost incurred to the fungus by the endosymbiont. Hence, we conclude that the type of endosymbiont in the fungus can have a direct effect on the tolerance of the fungi to metals.

In the case of the effects of types of metals, for non-essential metals (such as Cr^6+^, Cu^2+^, and Pb^2+^), compared to essential metals (Ca^2+^, Fe^2+^, and Mn^2+^), the former was responsible for greater fungal inhibition. The inhibitory effect of these metals was particularly evident when we considered Cu^2+^ and Cr^6+^. For both *Linnemannia* and *Benniella* with or without endobacteria, for the concentrations tested, the presence of Cu^2+^ (0.5 mM) and Cr^6+^ (1 mM) did not affect the metal tolerance index. This demonstrates that the endosymbionts will not always benefit the host for all types of toxic metals. Hence the toxicity can be related to the type of metal, not necessarily to the presence or absence of the endosymbiont. On the other hand, when the metal is less toxic or beneficial (essential metal) for the microorganisms, the effect due to the presence of the bacteria will be more visible, increasing, in the case of *Benniella*, or lowering, in the case of *Linnemannia*, the tolerance toward the metals. For instance, for both *Linnemannia* and *Benniella* with or without endobacteria, in the presence of Ca^2+^, the growth increased compared to the control, *i.e.*, the fungi without the metal. This result might be explained by the fact that an increased concentration of calcium in the cytosol was associated with fungal growth through hyphal elongation (Juvvadi et al., 2011; Hu et al., 2014) and cell cycle progression (Nanthakumar et al., 1996; Miyakawa and Mizunuma, 2007). A similar result was also noticed for *Linnemannia* in the presence of Mn^2+^, where both *Linnemannia* + and *Linnemannia* -, with no statistically significant difference between the two, had a noticeable increase in growth compared to the control without the metal. This can be explained by the fact that Mn^2+^ can be a limiting factor for the fungal metabolism (Manikan et al., 2014), and an increased concentration available for the fungus could have increased fungal growth.

Our results show that in the case of highly toxic metals, such as Cu^2+^ and Cr^6+^, the influence on MIC and tolerance index due to the presence of the endobacterium was negligible. On the other hand, when the toxicity of the metal decreased, the contribution of the endobacterium toward the fungus resistance became more evident and therefore dependent on the type of interaction existing between the host and the symbiont.

### The Effect of Endosymbiont on the Fungal Metal Biosorption Properties

Fungi and bacteria, in recent years, have been considered for their ability to remove metals as a sustainable alternative for metal remediation (Fan et al., 2014; Mejias Carpio et al., 2018). To verify whether the metal removal was attributable to adsorption or active metabolic processes, experiments were carried out on dead and live fungi. Fungi are in fact capable of uptake metal ions through transport channels (Ohsumi and Anraku, 1981; White and Gadd, 1987; Cohen et al., 2000), low-affinity permeases of divalent metal ions (Nelissen et al., 1997; Kosman, 2003), or non-specific metalloreductase (Kosman, 2020) and also of carrying out metabolic activities aimed at reducing the toxicity of the metals present, e.g., chelation or translocation of the metal (Ahmad et al., 2005). Unlike living mycelium, the dead biomass can only carry out adsorption on the surface of the mycelium.

In general, we have noticed that the presence of endobacteria, both for *Benniella* and *Linnemannia*, was not linked to greater removal of metals. Both dead *Benniella*+ and dead *Benniella* – removed high concentrations of Cu^2+^, Cr^6+^, Pb^2+^, and Fe^2+^ compared to living mycelium, which suggests that adsorption can be happening. In the case of dead *Linnemannia* + and dead *Linnemannia* -, a greater removal of Cr^6+^, Pb^2+^, Ca^2+^, and Fe^2+^ was observed compared to live biomass. These results demonstrate that the removal of non-essential metals (e.g., Cr^6+^, Pb^2+^) and essential metals (e.g., Ca^2+^, and Fe^2+^), for both *Linnemannia* and *Benniella*, can be largely attributed to adsorption (Lilly et al., 1992; Butter et al., 1998) and that the live fungi are actively putting in place mechanisms aimed at reducing the uptake of the metal. Furthermore, we also observed that the metal removal performed by the dead fungus was less affected by the presence or absence of bacteria when compared to the respective live fungus. This result might indicate that the bacteria, when present in the fungus, could also influence the metal uptake activity once the metal ions have entered the fungus.

In addition to the comparison between the metal removal performed by dead and live biomass, we determined whether the presence of endobacteria could affect metal removal in the live biomass. In general, we observed that the presence of endobacteria, both BRE and MRE, was not linked to higher removal of metals except for Fe^2+^ for the fungus *Benniella*+ and Ca^2+^ for the fungus *Linnemannia*+. These results could be because the endosymbionts, localized in the vicinity of lipid bodies, were responsible for a reduction in the number of these lipid-rich organelles compared to the cured fungus (Uehling et al., 2017; Desirò et al., 2018). A reduction in lipid bodies, which can be used as storage for potentially toxic compounds, such as heavy metals (Clark and Zeto, 2000; Fayeulle et al., 2014), could explain how the cured fungi led to a generally greater removal of metals.

Therefore, these results demonstrate that for non-metal resistant fungi, such as those selected in this study, the main method of metal removal is due to adsorption by the hypha. Additionally, the presence of endobacteria, which affect the composition of the lipid bodies associated with the fungus, may have reduced the metal uptake by the host.

### Effect of Different Metals on the Abundance of Endosymbionts

Bacteria are known for their ability to resist and accumulate metals present in the external environment. Currently, there is still a vast knowledge gap regarding how this ability is maintained when the bacterium has established a symbiotic interaction within the fungal hypha. To shed light on this knowledge gap, different concentrations of essential and non-essential metals were tested in this study to determine their influence on the presence of endobacteria.

Our results show that, although not statistically significant, the abundance of BRE and MRE are characterized by two different trends. In the case of *Benniella*+, the relative abundance of the bacterium increased when the fungus was exposed to a higher concentration of the metal (Cu^2+^, Cr^6+^, Ca^2+^, Mn^2+^), while for *Linnemannia*+, an increase in the concentration of the metal coincided with a reduction in the abundance of the endobacteria (Cu^2+^, Cr^6+^, Pb^2+^, Fe^2+^, Mn^2+^). This result could be linked to the fact that, as noticed by the tolerance index, in the case of *Benniella* +, the endobacteria contributes to facilitating the resistance of the host to different metals. Also, in the case of this fungus, for metals in which the presence of the endobacteria increased the tolerance index (Cr^6+^, Ca^2+^, and Mn^2+^), we found a linear correlation between the metal removal ratio between *Benniella*+ and *Benniella* –, and the relative abundance of 16S rRNA gene (Supplementary Figure 7).

As for the fungus *Linnemannia*+, as observed from the MIC and tolerance index results, increasing concentration of the endobacteria did not coincide with an increase in the metal removal ratio in *Linnemannia*+ compared to *Linnemannia* –. This result could be explained by the fact that, because of the stress ratio to which the fungus is subjected, this has led to exacerbating the energy deficit sustained for the maintenance of the bacterium (Wernegreen, 2012; Bastías et al., 2020). It is in fact suspected that in symbiotic relationships, there may be a modulation of the flow of nutrients from the host to the symbiont to consequently control the growth rate of the latter (González-Guerrero et al., 2016). Interestingly, for both fungi, we determined that the relative abundance of the bacteria in the presence of the metal was in general higher compared to the control. This result could be explained by the fact that the endobacteria is subjected only to a portion of metals that cross the fungal cell wall, leading to a change in the growth ratio between the host and symbiont.

The results show that depending on the type of host-symbiont system, the fungus can perform a potential modulation of the endobacteria and, in the case of *Benniella*+, there is a dependency between the abundance of the symbiont and metal removal.

### Interactions of the Fungal and Bacterial-Fungal Functional Groups With Different Metals

The uptake of heavy metals by fungi may impact complex metabolic processes, which can cause various morphological modifications, including the reduction of growth, alteration of the structure of the mycelium (Baldrian, 2003), and the modification of the composition of the membrane (Howlett and Avery, 1997). These effects are caused by the powerful inhibitory action of heavy metals against enzymes, cell membranes, and organelles (Vallee and Ulmer, 1972), which can lead to oxidative stress (Stohs and Bagchi, 1995). Fungi are able, through the use of different methods (e.g., valence transformation, intra- and extracellular precipitation, uptake and translocation into lipid bodies, complexation with chelators, and low molecular weight peptides), to reduce the metal toxicity and improve tolerance (Tomsett, 1993; Clark and Zeto, 2000; Zafar et al., 2007; Fayeulle et al., 2014).

The variation in the functional groups associated with essential and non-essential metal treatments in the presence or not of endobacteria was determined by ATR-FTIR analysis. This analysis has allowed us to determine the possible structural modification that could be occurring in the two fungi studied, which can be associated to the presence of metals or the endobacteria. HCA was used as a tool to determine the distances between the samples spectra and determine possible clusters attributable to the presence/absence of endobacteria and the type of metal. In order to determine which single or multiple functional groups may have been influenced by the presence of the metal treatment and presence/absence of endobacteria, the different peaks were analyzed individually (Figures 6, 7 and Supplementary Table 1).

Based on the comparison of the spectra obtained from the controls and the metal-exposed fungi, we noticed that the difference between the individual functional groups of *Benniella*+ and *Benniella* – was mainly attributable to the type of metal. The results were also confirmed by HCA (Figure 8) where we could observe that the distance calculated between the samples shows how the different isogenic lines, *Benniella*+ and *Benniella* –, are strongly similar to each other while they differ based on the type of metal exposure. We also noticed that there were two separate clusters for *Benniella* splitting essential metals (Fe^2+^, Ca^2+^, and Mn^2+^) from non-essential metals (Cu^2+^, Cr^6+^, Pb^2+^). Demonstrating that the types of metals can play a more important role in the functional groups expressed in the fungi.

Regarding the effect induced by the treatment with metals, we can see that *Benniella* –, when compared to *Benniella*+ in the presence of Ca^2+^ and Cu^2+^, triggered an increase in the intensity of the peaks related to the C-H asymmetric stretching (2,924 and 2,853 cm^–1^) and carbonyl group (1,745 cm^–1^) (Figure 6). Previous study has shown that the curation of the fungus from its endosymbiont could lead to an increase in the number of lipidic bodies compared to the WT fungus (Uehling et al., 2017). The increase of these functional groups could also be caused by oxidative stress on the cell membrane wall, as evidenced by the peak at 1,742 cm^–1^ related to the carbonyl group from the ester. This particular peak is typically generated after the peroxidation of fatty acids (Fuchs et al., 2011; Oleszko et al., 2015). The oxidation of lipids is known to increase in the presence of transition metals (Cu^2+^, Cr^6+^, Fe^2+^, and Mn^2+^) (Zschornig et al., 2004) and post-transitional metal (Adonaylo and Oteiza, 1999). Moreover in the presence of reactive oxygen species (ROS), it is hypothesized that these metals can also act as catalysts in the decomposition process of hydrogen peroxide (Fenton reaction) (Oteiza et al., 2004; Repetto et al., 2010). Additionally, in the presence of Fe^2+^ and Mn^2+^, the *Benniella*+ fungus appears to have a reduction in the intensity of the same peaks, suggesting a lower susceptibility to these metals. This hypothesis is also sustained by the tolerance index (Figure 2) where, except for Fe^2+^, it is evident that the *Benniella* + is less inhibited by the presence of these metals.

*Linnemannia elongata*, on the other hand, from the HCA (Figure 9) results, shows the importance of the presence of endobacteria rather than the type of metal, since two clusters were formed. One of the clusters contained the fungi with the presence of endobacteria and the other one was the cured fungi. The presence of endobacteria caused clear changes in the functional groups present on the surface of the fungus. From the ATR-FTIR data, it emerged that in the presence or not of the different metals, the fungus *Linnemannia* –, when compared to *Linnemannia*+, presented a marked increase in the peaks related to lipidic (2,924 and 2,853 cm^–1^) and protein (1,742 and 1,544 cm^–1^) regions. As previously described, the endobacteria elimination from the fungal host led to an increased number of lipidic bodies compared to the WT fungus (Uehling et al., 2017). These lipid bodies are used for the compartmentalization of pollutants as a form of defense mechanism (Lenoir et al., 2016). In addition to the increase in lipidic and protein-related peaks, in the presence of Cu^2+^, Fe^2+^, and Mn^2+^ for *Linnemannia* –, it was also observed that there was an increase in the intensity of the peaks at 1,150 and 1,077 cm^–1^ corresponding to phosphate and sulfide groups. These negatively charged functional groups are known to take part in the physicochemical process of cell adsorption (Dhankhar and Hooda, 2011; Pugazhendhi et al., 2018; Chen et al., 2019). These results appear to agree with the data related to the tolerance index (Figure 2), MIC (Figure 1), and metal removal (Figure 4), where we were able to notice not only an overall higher tolerance of *Linnemannia* – compared to *Linnemannia*+, especially toward Fe^2+^ and Mn^2+^, but also an increase in metal removal.

In general, the functional groups that exhibited intensity changes could potentially be involved in the adsorption process of the metals by these fungi. In this study, the major functional group changes were the C-H stretching from the lipids (2,924 and 2,853 cm^–1^), amide functional group from the proteins (1,742 cm^–1^), and to a lesser extent the symmetrical and asymmetrical stretching of PO_2_^–^ and P (OH)_2_ at 1,150, 1,077, and 1,026 cm^–1^.

In conclusion, through this research, we have characterized the impact of endosymbiotic bacteria on the response and uptake of essential and non-essential metals by non-metal-resistance in early diverging fungi in Mortierellaceae. The results showed that the response toward essential and non-essential metals is mainly driven by the type of endobacteria colonizing the fungal mycelium. For *L. elongata* (*Linnemannia*), the presence of *Burkholderia*-related endobacterial symbiont was detrimental; in fact, curation of the fungi from the endobacteria led to increasing fungal tolerance to different metals. Curing *L. elongata* also influenced the physicochemical composition of the functional groups present on the mycelium to allow the fungus to tolerate different metals (Cr^6+^, Pb^2+^, Ca^2+^, and Fe^2+^). Furthermore, the presence of the endosymbiont did not lead to an appreciable increase in uptake of the metals.

On the other hand, *B. eronia* (*Benniella*) benefited from the presence of the endobacteria by increasing the fungal tolerance to Cr^6+^, Pb^2+^, Ca^2+^, and Mn^2+^, which was demonstrated by a positive correlation between endobacterium abundance and relative metal removal. This study provides a broad view on how the response toward different metals, whether essential or non-essential, is influenced by the type of fungus-bacterium association and, more specifically, by the fact that the maintenance of the endobacteria can have a metabolic cost for the host in certain cases. It is also possible that lipids play an important role in the fungal defense against metal pollutants and that the endobacteria in the fungal microbiome can potentially affect their composition and consequently affect the host resistance to metals. This study, therefore, informs future studies on fungal endobacteria and underlying mechanisms in the resistance and uptake of different metals from the environment.

To answer this question, various metals were selected based on their known cell toxicity (Cu^2+^, Cr^6+^, and Pb^2+^) and essentiality for biological processes (Ca^2+^, Mn^2+^, and Fe^2+^). This question was addressed using an interdisciplinary approach, to evaluate the biological and physicochemical aspects associated with the presence of the endobacterium within the fungus.

The results show that the type of host–symbiont association can alter the resistance of the fungal host and modulate the functional groups expressed and exposed on the hyphae in the presence of metals. This study is, therefore, an initial step in evaluating the potential functionalities of endobacteria associated with fungi.

## Data Availability Statement

The original contributions presented in the study are included in the article/Supplementary Material, further inquiries can be directed to the corresponding author/s.

## Author Contributions

DR, GB, and SL contributed to the conception and design of the study. GB provided the cured and wild-type strains for the study. DR contributed to data validation and interpretation, overall manuscript writing and editing, resources for the execution of the project, supervision, administration of the overall project, and funding acquisition. SL and JP-B performed the experiments, data collection, data analysis, and data visualization. SL wrote most parts of the manuscript. JP-B wrote the ATR-FTIR section of the manuscript and assisted in the overall manuscript editing. All authors contributed to manuscript revision and approved the submitted version.

## Conflict of Interest

The authors declare that the research was conducted in the absence of any commercial or financial relationships that could be construed as a potential conflict of interest.

## Publisher’s Note

All claims expressed in this article are solely those of the authors and do not necessarily represent those of their affiliated organizations, or those of the publisher, the editors and the reviewers. Any product that may be evaluated in this article, or claim that may be made by its manufacturer, is not guaranteed or endorsed by the publisher.
