# Supplementary material for: Effect of Endosymbiotic Bacteria on Fungal Resistance Toward Heavy Metals
Source: Front Microbiol. 2022 Mar 15;13:822541. doi: 10.3389/fmicb.2022.822541 (PMC8965456; doi:10.3389/fmicb.2022.822541)
Supplement: Supplementary file 1 [file Data_Sheet_1.docx]

Effect of endosymbiotic bacteria on fungal resistance towards heavy metals

Simone Lupini^1,2^, Janire Pena-Bahamonde^1^, Gregory Bonito^3^, Debora F. Rodrigues^1,2^*

^1^Department of Civil and Environmental Engineering, University of Houston, Houston, TX 77004, USA

^2^Department of Biology and Biochemistry, University of Houston, Houston, TX 77004, USA

^3^College of Agriculture & Natural Resources, Department of Plant, Soil and Microbial Sciences, Michigan State University, Michigan, MI 48824

*** Correspondence:**Corresponding Author
dfrigirodrigues@uh.edu (D.F. Rodrigues)

Figure S1: Amplification plot of the bacterial 16S rRNA gene obtained from the **A)** serial dilution of the standard and the samples **B)** Benniella and **C)** Linnemannia. The red line indicates the threshold used to determine the Ct values.

Figure S2: Standard curve of the bacterial 16S rRNA gene obtained from the serial dilution of the standard. The value of Ct was plotted against the copy number (log copies).

Figure S3: ATR-FTIR spectra of the fungus A) Benniella + and B) Benniella - with different metals (Cu^2+^, Cr^6+^, Pb^2+^, Ca^2+^, Fe^2+^, and Mn^2+^).

Figure S4: ATR-FTIR spectra of the fungus A) Linnemannia + and B) Linnemannia - with different metals (Cu^2+^, Cr^6+^, Pb^2+^, Ca^2+^, Fe^2+^, and Mn^2+^).





Figure S5: Principal component analysis of the ATR-FTIR spectra of Benniella + and Benniella -.





Figure S6: Principal component analysis of the ATR-FTIR spectra of Linnemannia + and Linnemannia -.

Table S1: Relative peak intensities for A) Benniella + (GBA WT), B) Benniella - (GBA cur), C) Linnemannia + (NVP WT) and D) Linnemannia – (NVP cur) at different wavenumbers (2924, 2853, 1742, 1637, 1544, 1150, 1077, 1027 cm^-1^). The values reported are calculated based on the ratio between the relative intensity of the fungus in the presence of the metal with respect to the fungus.

| 1. ***Benniella* +** | **Relative peak intensity** | | | | | |
| --- | --- | --- | --- | --- | --- | --- |
| **Wavenumbers (cm^-1^)** | **Cu^2+^** | **Cr^6+^** | **Pb^2+^** | **Ca^2+^** | **Fe^2+^** | **Mn^2+^** |
| ν _s (C-H)_= **2924** | 2.02 | 1.31 | 0.51 | 1.42 | 0.55 | 0.95 |
| ν _as (C-H)_ **= 2853** | 2.22 | 1.51 | 0.54 | 1.57 | 0.47 | 0.90 |
| ν _C=O_ = **1742** | 3.30 | 1.86 | 0.37 | 2.02 | 0.27 | 1.00 |
| δ **_N-H_ = 1637** | 1.29 | 0.88 | 0.60 | 1.32 | 0.81 | 0.91 |
| δ **_N-H_ = 1544** | 1.42 | 0.89 | 0.48 | 1.29 | 0.78 | 0.86 |
| ν _C-O_ **= 1150** | 0.94 | 0.99 | 0.74 | 1.01 | 0.65 | 1.01 |
| ν _s_ (PO_2 −),_  ν (PO_4_ ^2−^) , ν _C−O−C_, and ν _C−O−P_ = **1077** | 1.08 | 1.09 | 1.08 | 1.26 | 1.04 | 1.16 |
| ν _C-O_ **= 1027** | 1.00 | 1.02 | 1.00 | 0.99 | 0.99 | 0.99 |

| 1. ***Benniella* -** | **Relative peak intensity** | | | | | |
| --- | --- | --- | --- | --- | --- | --- |
| **Wavenumbers (cm^-1^)** | **Cu^2+^** | **Cr^6+^** | **Pb^2+^** | **Ca^2+^** | **Fe^2+^** | **Mn**^2+^ |
| ν _s (C-H)_ **= 2924** | 1.54 | 1.56 | 0.92 | 4.05 | 0.69 | 0.66 |
| ν _as (C-H)_ **= 2853** | 1.61 | 1.61 | 0.98 | 3.89 | 0.75 | 0.62 |
| ν _C=O_ = **1742** | 1.97 | 1.72 | 0.96 | 4.47 | 0.53 | 0.31 |
| δ **_N-H_ = 1637** | 1.07 | 1.04 | 0.74 | 1.89 | 0.91 | 1.02 |
| δ **_N-H_ = 1544** | 0.98 | 1.12 | 0.66 | 1.97 | 0.96 | 0.96 |
| ν _C-O_ **= 1150** | 0.95 | 1.17 | 0.88 | 1.12 | 0.87 | 1.13 |
| ν _s_ (PO_2 −),_ ν (PO_4_ ^2−^), ν _C−O−C_, and ν _C−O−P_ = **1077** | 0.96 | 0.99 | 0.99 | 1.09 | 1.00 | 1.04 |
| ν _C-O_ **= 1027** | 1.00 | 1.02 | 1.00 | 0.99 | 0.99 | 0.99 |

| 1. ***Linnemannia* +** | **Relative peak intensity** | | | | | |
| --- | --- | --- | --- | --- | --- | --- |
| **Wavenumbers (cm^-1^)** | **Cu^2+^** | **Cr^6+^** | **Pb^2+^** | **Ca^2+^** | **Fe^2+^** | **Mn^2+^** |
| ν _s (C-H)_ **= 2924** | 1.84 | 1.63 | 3.12 | 7.60 | 1.75 | 1.33 |
| ν _as (C-H)_ **= 2853** | 0.56 | 0.47 | 1.05 | 2.67 | 0.26 | 0.20 |
| ν _C=O_ = **1742** | 0.26 | 0.28 | 1.63 | 4.15 | 0.17 | 0.20 |
| δ **_N-H_ = 1637** | 1.20 | 0.70 | 1.15 | 0.74 | 1.54 | 1.30 |
| δ **_N-H_ = 1544** | 2.12 | 0.83 | 2.18 | 1.21 | 2.60 | 2.04 |
| ν _C-O_ **= 1150** | 5.14 | 6.27 | 7.09 | 13.04 | 8.05 | 9.00 |
| ν _s_ (PO_2 −),_ ν (PO_4_ ^2−^), ν _C−O−C_, and ν _C−O−P_ = **1077** | 1.16 | 1.16 | 1.24 | 1.20 | 1.28 | 1.46 |
| ν _C-O_ **= 1027** | 1.20 | 1.23 | 1.23 | 1.22 | 1.17 | 1.16 |
| 1. ***Linnemannia* -** | **Relative peak intensity** | | | | | |
| **Wavenumbers (cm^-1^)** | **Cu^2+^** | **Cr^6+^** | **Pb^2+^** | **Ca^2+^** | **Fe^2+^** | **Mn^2+^** |
| ν _s (C-H)_ **= 2924** | 2.34 | 0.86 | 1.05 | 1.52 | 4.10 | 3.56 |
| ν _as (C-H)_ **= 2853** | 3.02 | 1.00 | 1.07 | 1.97 | 5.20 | 4.50 |
| ν _C=O_ = **1742** | 3.02 | 0.77 | 1.01 | 1.53 | 5.44 | 4.24 |
| δ **_N-H_ = 1637** | 0.75 | 0.22 | 1.01 | 0.53 | 0.96 | 1.02 |
| δ **_N-H_ = 1544** | 0.71 | 0.16 | 1.04 | 0.47 | 0.90 | 1.07 |
| ν _C-O_ **= 1150** | 1.45 | 0.90 | 1.02 | 1.08 | 1.80 | 1.76 |
| ν _s_ (PO_2 −),_ ν (PO_4_ ^2−^), ν _C−O−C_, and ν _C−O−P_ = **1077** | 1.01 | 0.82 | 1.01 | 0.97 | 1.06 | 1.23 |
| ν _C-O_ **= 1027** | 1.13 | 1.10 | 1.02 | 1.14 | 1.08 | 1.21 |

Figure S7: Linear fit of the metal removal ratio between Benniella + and Benniella - and the relative abundance of the 16S rRNA for the metals in which the presence of endobacteria has increased tolerance index (Cr^6+^, Pb^2+^, Ca^2+^, and Mn^2+^).
